# The Side-Effect Effect in Children Is Robust and Not Specific to the Moral Status of Action Effects

**DOI:** 10.1371/journal.pone.0132933

**Published:** 2015-07-28

**Authors:** Hannes Rakoczy, Tanya Behne, Annette Clüver, Stephanie Dallmann, Sarah Weidner, Michael R. Waldmann

**Affiliations:** Institute of Psychology & Courant Research Centre, Evolution of Social Behaviour, University of Göttingen, Waldweg 26, D- 37073 Göttingen, Germany; University of New South Wales, AUSTRALIA

## Abstract

Adults’ intentionality judgments regarding an action are influenced by their moral evaluation of this action. This is clearly indicated in the so-called *side-effect effect*: when told about an action (e.g. implementing a business plan) with an intended primary effect (e.g. raise profits) and a foreseen side effect (e.g. harming/helping the environment), subjects tend to interpret the bringing about of the side effect more often as intentional when it is negative (harming the environment) than when it is positive (helping the environment). From a cognitive point of view, it is unclear whether the side-effect effect is driven by the moral status of the side effects specifically, or rather more generally by its normative status. And from a developmental point of view, little is known about the ontogenetic origins of the effect. The present study therefore explored the cognitive foundations and the ontogenetic origins of the side-effect effect by testing 4-to 5-year-old children with scenarios in which a side effect was in accordance with/violated a norm. Crucially, the status of the norm was varied to be conventional or moral. Children rated the bringing about of side-effects as more intentional when it broke a norm than when it accorded with a norm irrespective of the type of norm. The side-effect effect is thus an early-developing, more general and pervasive phenomenon, not restricted to morally relevant side effects.

## Introduction

Decades of research on theory of mind and moral reasoning have produced considerable insight into the development of the cognitive processes underlying each of these two social-cognitive domains. But there has been comparatively little systematic research on their functional relation [1–5]. The exception is research on the influence of perceived intentionality behind an action on children’s moral judgments of the corresponding act. This research has shown that during the preschool years children’s moral judgments begin to be informed and modulated by information about the agent’s intentionality. Children this age, for example, begin to excuse acts with bad consequences when they are performed with good intentions or distinguish between purely accidental mistakes and negligent ones [6–12].

Recent experimental work with adults has also addressed the question of how perceived intentionality and moral judgments are interconnected. Interestingly, this research has uncovered less commonsensical and more surprising relations between the two domains, namely influences going in the opposite direction from moral evaluation to theory of mind judgment. Most prominent in this respect is the so-called ‘side-effect effect’ (SEE). When told about an action with an intended main effect and a merely foreseen side effect, subjects tend to interpret the bringing about of the side effect more often as intentional when it is morally negative than when it is positive [13–16]. In a typical vignette, subjects hear about the chairman of a company thinking about starting a new program as a means to achieve more profit, the primarily intended effect. When informed by the vice president that the program would help/harm the environment as a side effect, the chairman replies that he does not care about the environment but only about profits. He decides to implement the program which in fact helps/harms the environment. When then asked “Did the chairman help/harm the environment intentionally?”, subjects answer “yes” significantly more often in the harming than in the helping case [15].

How best to interpret and explain this effect is currently a much debated and very controversial issue [17,18]. Two questions discussed in this context are of particular relevance for the present study. The first is whether or not the side-effect effect is a pervasive cognitive phenomenon. Some researchers have doubted that the SEE shows us much about our social cognition, suggesting instead that it simply reflects specific task pragmatics regarding how to interpret the presented linguistic material. Most prominently, it has been argued that the test question (whether the chairman brought about the foreseen side effect intentionally), though from a semantic point of view just dealing with intentionality, pragmatically evokes conversational implicatures to the effect that it is actually questions of guilt and blame that are at issue [19]. According to this interpretation, subjects do not answer the question at the semantic level and engage in intentionality ascription, but in fact they answer a different, implicated question, namely whether the chairman is to be blamed for bringing about the side effect. Other accounts, in contrast, have claimed that, given that the SEE has been documented across different cultures and in different contexts, it reflects a fundamental and pervasive design feature of our social-cognitive makeup [18,20]. The second question concerns the content specificity of the SEE. Some accounts consider it as, essentially, a phenomenon of *moral* judgment and its (non-rational) influence on theory of mind [21,22]. In contrast, other accounts suggest that the SEE might be a more general phenomenon, having to do with normative judgments more broadly, not only with moral ones [13]. *Rationality accounts*, in particular, view the SEE as a manifestation of a rational strategy of diagnosing mental states. One version of such an account claims that actions violating a norm are generally more diagnostic of the actor and her motives and traits than actions in accordance with norms. Whereas actions conforming with norms are ambiguous because they can be explained either by the agent’s intentions or by societal norms, deviations from the norm are more diagnostic for an intention, [13,17]. Recent empirical findings with adults, in fact, are consistent with the latter possibility: the same SEE answer patterns could be found, for example, when the side effect was or was not in accordance with a purely arbitrary conventional rule without any obvious moral relevance [17].

To resolve both of these questions-regarding the cognitive pervasiveness of the SEE and its content-specificity—developmental data about the ontogenetic roots and foundations of the side-effect effect are highly relevant. The earlier the SEE can be found in ontogeny, in particular at an age at which children are not yet susceptible to conversational implicatures in adult-like ways [23], and the bigger the variety of domains for which the SEE can be documented (conversational implicatures in terms of blame might be plausible in moral domains, but not or much less in conventional ones) the less likely it seems that it is just a marginal artifact of adult task pragmatics; and as a consequence, the more justified the conclusion becomes that the SEE reflects a substantial and pervasive feature of our social cognition.

So far, however, little is known about the ontogenetic foundation of the side-effect effect: first of all, to date there have only been three studies at all on the SEE in children, all of which are difficult to interpret given their methodological deviations from the adult studies [24–26]. The adult experiments carefully matched the positive (help the environment) and negative conditions (harm the environment) in all respects except the valence of the side effect [15]. The three existing studies testing for the SEE in children, in contrast, confounded the difference between negative and positive valence with a difference in the character’s attitude vis-à-vis the side effect: In the prototypical vignette in these studies a boy loves frogs and therefore brings a frog over to a girl’s house who (as he knows) will be happy (positive) or upset (negative) about the frog. The boy’s attitudes towards the side effect were then described differently in the two conditions:

- The boy does not care that the girl will be happy. He is going to bring the frog over *just for himself* (positive)
- The boy does not care that the girl will get upset. He is going to bring the frog over *anyway* (negative)

Children from age 4–5 showed the side-effect effect in these studies. This finding, however, is very difficult to interpret given the confound between the valence of the side effect and the actor’s attitude towards it. In the adult studies with their minimal contrast design (positive and negative conditions differed only in the valence of the side effect), the interpretation that it must have been the moral status of the side effect that made the difference–and that thus the side effect effect is rightly called by this name- is conclusive. In the existing child studies, in contrast, we do not know what made the difference: the moral status of the side effect, the difference in how the agent’s attitude toward the side effect was described, or both? This is potentially troublesome since it is intuitively plausible that the two descriptions might have carried rather different implications: “just for himself” might have emphasized the fact that the protagonist did not even think about anything apart from his own interests, whereas “anyway” might have licensed the pragmatic inference that the protagonist was well aware of others’ concerns (the girl’s aversion to frogs and her getting upset) but deliberately chose not to take them into account. This would naturally lead to an evaluation of the negative side effect as a case of negligence (others’ interests are considered but not respected) and as such as more intentional that the bringing about of the positive side effect in which others’ interests were not even an issue [for a treatment of such pragmatic factors in side-effect effect tasks, see, e.g., 19]. As a consequence, we do not know whether children actually show the side-effect effect, or whether their answer pattern found in previous studies merely reflect other differences (in the description of the agent’s attitudes) confounded with moral valence.

Second, in addition to the uncertainty whether the SEE is real in children in the first place, even if it turns out to be a robust phenomenon, we do not know how specific it is to *morally* relevant side effects. No developmental study has so far explored side-effect effects with violations of norms other than moral ones at all.

The aim of the present study was therefore twofold: First, we set out to test whether the side-effect effect is a real, reliable and robust phenomenon in children, at all. To do so, we tested children at the youngest age at which SEE-tasks seem to be reasonably applicable. SEE tasks, measuring children’s liability to be influenced in their explicit intentionality judgments by the normative status of a side effect, presuppose a solid capacity to answer explicit intentionality questions in the first places. Such a capacity has been documented from around 4–5 years: children this age correctly ascribe intentions to others and themselves, distinguishing, for example, intentions from mere desires, and contrasting unintentional yet successful behavior from true intentional success [27–34]. Following up on existing studies, we tested for the SEE in children by using a more stringent method: we removed previous confounds (by keeping constant how the agent’s attitude towards the side effect was described) and administered a minimal contrast pair between positive and negative side effects with the only difference being the side effect’s valence.

The second aim was to investigate the normative specificity of any potential SEE in children. We therefore systematically tested for the SEE using side effects violating either moral or non-moral norms. As in the pioneering adult experiments by Uttich and Lombrozo (2010), we contrasted paradigmatic cases of (what are commonly taken as) moral norms and paradigmatic cases of (what are commonly taken as) arbitrary conventional norms without any obvious moral element or relevance. For the former we used cases in which a recipient was affected emotionally in positive/negative ways by the side effect (made happy/upset), and for the latter we used cases with an arbitrary rule (e.g. as to where certain animals or vehicles should/should not be or go). In a 2x2 design, children were presented with closely matched vignettes in which a protagonist performed an action with an intended primary effect (letting one animal out of a cage) and a foreseen side effect (another animal exiting the cage as well). The causal structure–the action and its relation to intended primary and foreseen side effect- were identical in the moral and conventional conditions. What varied between the moral and conventional cases was the kind of normative status: In the moral conditions, the side effect had moral relevance in that it pleased or frightened someone. In the conventional conditions, the side effect was/was not in accordance with a arbitrary conventional norm (to the effect that animals of this type ought to be/ought not to be in cages). In all condition, children were then asked whether the protagonist had brought about the side effect (letting the other animal out of the cage) intentionally.

## Method

### Participants

Fifty-four 4-5-year-old children participated in this experiment (29 girls, age range = 53–62 months, *M* = 57). The children were recruited from the Department of Developmental Psychology’s databank of children whose parents had previously given written consent to experimental participation, and came from mixed socio-economic backgrounds. Children were tested by one of three experimenters in their local daycare in an urban area in Germany. Eleven additional children were tested but excluded from the analyses due to consistent failure to answer control questions correctly (*n* = 4, see below for more details) or to give a clear “yes/no” reply to test questions (*n* = 3), due to experimental error (*n* = 3), or due to uncooperativeness (*n* = 1).

#### Ethics Statement

This research was conducted in accordance with the Declaration of Helsinki and the Ethical Principles of the German Psychological Society (DGPs), the Association of German Professional Psychologists (BDP), and the American Psychological Association (APA). It involved no invasive or otherwise ethically problematic techniques and no deception (and therefore, according to National jurisdiction, did not require a separate vote by a local Institutional Review Board; see the regulations on freedom of research in the German Constitution (§ 5 (3)), and the German University Law (§ 22)).

### Design and Procedure

A mixed 2x2 design was used with the valence of the side effect (positive vs. negative) as within subjects-factor and norm type (conventional vs. moral) as between-subjects factor. Each child, thus, received two conditions (positive and negative), with two tasks per condition, resulting in a session with four tasks. The task vignettes were shown to children as Power Point animations on a laptop computer. There were four story themes, each of which could be administered in the moral and the conventional conditions with positive and negative side effects (see S1 Table for details on the scripts). For example, in the mouse/bunny theme, the experimenter presented the following causal structure to the children: A mouse and a bunny were in a cage together. By opening the cage (means) one could let out the mouse (primary effect) to play with it, but incidentally then the bunny would also exit the cage (side effect). In the moral norm conditions, the fact that the bunny leaves the cage makes her owner happy (positive) or frightened (negative). In the conventional norm conditions, there was an arbitrary conventional rule regulating the bunny’s location (that bunnies ought to/ought not to be in cages), and letting out the bunny was either in accordance with that rule (positive) or constituted a violation of it (negative). Three control questions were administered to make sure children understood the scenarios. In the test question, children were asked whether the agent brought about the side effect intentionally (see Table 1 for details).

**Table 1:** Example of one scenario (mouse/bunny) in each of the 4 closely matched versions.

	Moral norm	Conventional norm
Introduction	--	“Listen, have you ever heard of Filla Land? They have many funny rules there. One of them is that bunnies are **supposed to/ not supposed to** run around the room.”
	“Look! Here you see mouse and bunny in a cage. Do you know what happens when you let the mouse out? Bunny automatically comes out too. Shall we take a look?” [*Pressing the space bar animates the slide*: *the cage door opens and both animals leave the cage*].“Did you see? Both animals left the cage.”	
Scenario	“Look! Here you see mouse and bunny in a cage. And this is Anna. Anna says: “I want to play with the mouse. I will let her out.”	
	--	“Anna’s sister says: “Here in Filla Land bunnies are **supposed to/ not supposed to** run around the room.”
**Side-Effect**	Anna’s sister says: “Anna, if you let the mouse out, the bunny automatically comes out too…	
	…. and baby Timmy is **happy/frightened**.”	… and will run all around the room.”
Control question 1	“What happens when Anna releases the mouse?”	
Control question 2	“And what happens with baby Timmy, when the bunny comes out?”	“And are bunnies supposed to run around the room?”
Indifference statement	Anna says “I don’t care what happens with baby Timmy. I just want to play with the mouse.”	Anna says “I don’t care what the bunny does. I just want to play with the mouse.”
Control question 3	“Does Anna care what happens with baby Timmy?”	“Does Anna care what the bunny does?”
**Result**	“Exactly, that’s why Anna goes ahead and releases the mouse. And then look, …	
	….baby Timmy is **happy/frightened**.”	…the bunny comes out too and runs around the room.”
**Test question**	“Baby Timmy was, **happy/frightened**, wasn’t he?…	“The bunny came out of the cage and is running around the room….
	… Did Anna do this intentionally?	

Each child received two blocks, each consisting of a trial with a positive and one with a negative side effect. Between the two test blocks, children completed some other task that was not part of the present study. Across children, the order of blocks and the within-block order were counterbalanced, as was the assignment of story themes to conditions. Each test block was only analyzed if a child had answered all control questions within that test block correctly. Thus, children who failed one or more control questions in each of their two blocks were excluded from the analyses (see *Participants* section).

## Results

**Fig 1:**
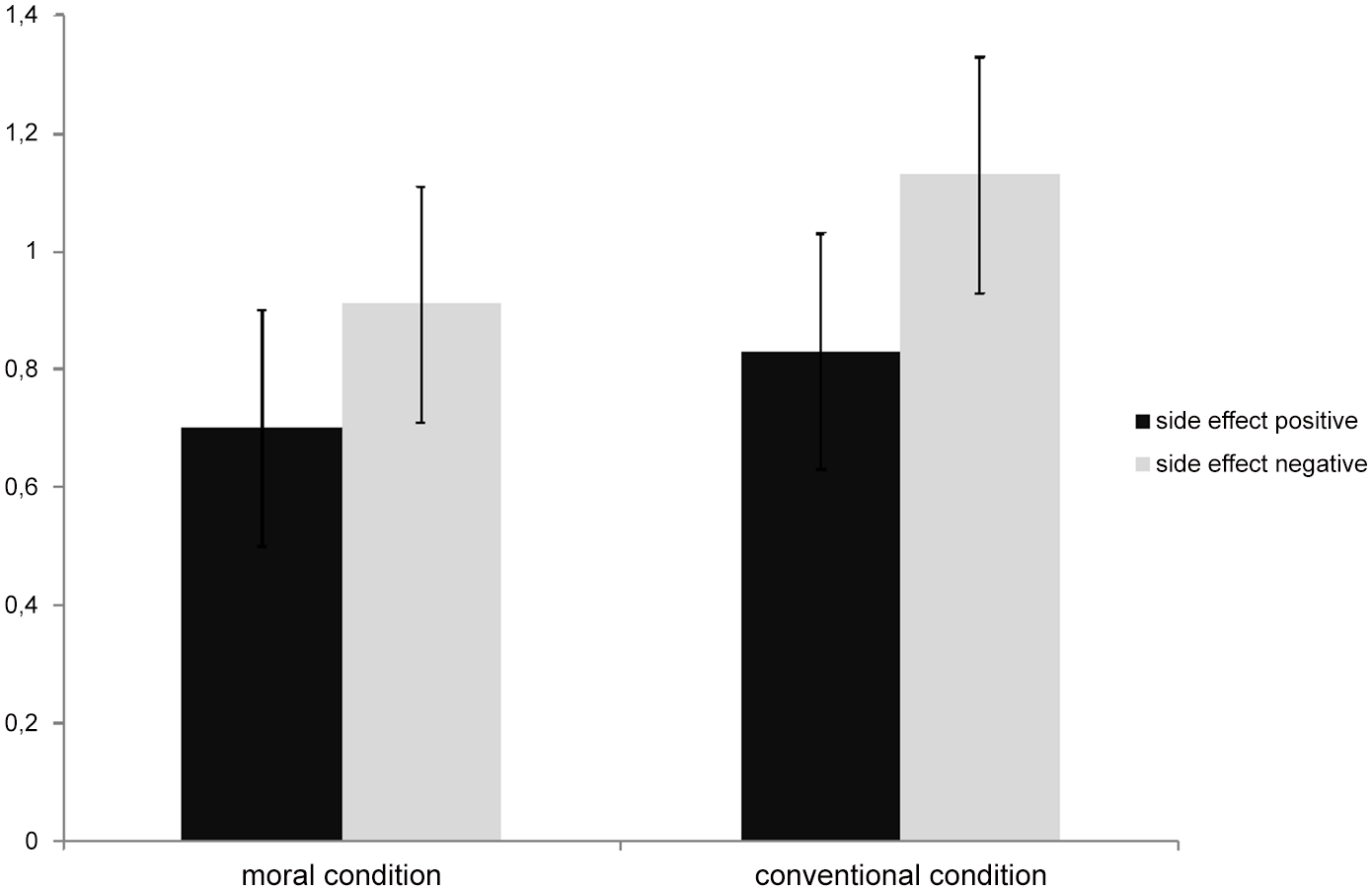
Mean number of trials (0–2) in which children said the side effect was brought about “intentionally” as a function of conditions.

The target dependent measure was children’s answers to the question whether the protagonist brought about the side effect intentionally in the four tasks each child received (2 positive/2 negative side effect). Of the 55 children in the final sample, 46 children answered the control questions to all 4 tasks correctly (23 in the moral and 23 in the conventional norm conditions). For these children, sum scores (0–2) were calculated for the two trials with the positive and for the two trials with the negative side effect summing the responses attributing intentionality to the agent. These data are depicted in Fig 1. A 2 (moral/conventional norm violation) x 2 (positive/negative valence of side effect) ANOVA on the mean number of trials in which children said the side effect was brought about “intentionally” revealed a significant main effect of valence: *F*(1, 44) = 6.63, *p*<.05, *η*_*p*_^2^ = .131 (with no effect of type of norm: *F*(1, 44) = .70, *p* = .51 and no interaction effect, *F*(1, 44) = .18, *p* = .67).

The eight children who wrongly answered control questions in one of their blocks of tasks gave similar responses. Given that for these children, sum scores could not be computed, we only analyzed the block (consisting of one task with positive and one with negative side effect) for which control questions were answered correctly. Three of these children showed the “side-effect effect” pattern by answering “intentionally” in the negative condition but “not intentionally” in the positive condition, while the others gave the same answer to the test question in both the negative and the positive condition (see Table 2).

**Table 2:** Contingency pattern (of those n = 8 children with only 1 valid test block) in the positive and negative side effect conditions.

		negative side effect	
		“intentionally”	“not intentionally”
positive side effect	“intentionally”	2	0
	“not intentionally”	3	3

## Discussion

Children in the present study rated foreseen side effects more often as intentional when they constituted a norm violation than when they did not. The present study is the first to document this under stringent experimental conditions in which the only relevant difference between the two cases was the valence of the side effect (positive/negative with regard to a norm). The side-effect effect thus seems to be a real and robust phenomenon already in children: their judgment of an action as intentional is influenced by whether or not the side effect, brought about by the action in question, violates a norm—even when potential confounding factors are removed. Furthermore, the side-effect effect in children is not confined to side effects that violate moral norms: the very same pattern held for side effects violating purely arbitrary conventional norms.

One interesting aspect of the present results in comparison to previous findings of the SEE in children is the following: even though the very same relative pattern of differences in the intentionality ratings between the positive and negative conditions were found across studies, children in the present study generally tended to be more conservative in their general intentionality ratings across all conditions (roughly 50% “yes” answers in the negative conditions compared to approximately 60–80% in comparable age groups in [24]). From the current findings alone we cannot tell why this might the case since the studies differ in various respects such as test language (here: German; previous studies: English or Italian) and the topics and structure of scenarios used (only the present study implemented a causal structure along the lines of adult vignettes, with a means aimed at a main effect and a foreseen side effect; see above). Most crucially, the studies differed in that the present study is the only child study with a minimal contrast design without confounds between positive and negative conditions in terms of the agent’s attitudes expressed. Future studies should systematically tease apart these different factors in order to investigate how they individually or jointly affect the general tendency to view the bringing about of foreseen side effects as intentional. More generally, it is an interesting question for future work, both in adults and from a developmental point of view, how the content, nature and strength of various norms might affect both the relative patterns of intentionality ratings (the SEE as such) and the absolute levels of intentionality judgments.

Notwithstanding these open questions, the present findings have several interesting implications: First, taken together with previous findings showing influences from intentionality ascription on normative judgment [e.g., 6,7], the present findings document influences in the other direction, and thereby suggest a systematic functional integration of the two social-cognitive domains-normative and theory of mind reasoning- from early on. This reciprocity is inconsistent with strong modularity claims to the effect that intentionality ascription and related forms of theory of mind cognition are modular, functionally encapsulated from other forms of social cognition such as normative evaluation.

Second, regarding the question of the cognitive basis of the SEE, the present findings suggest that the effect is not a late developing phenomenon emerging in adults but a more fundamental feature of our social-cognitive makeup observable early in ontogeny. Regarding the specificity of the SEE, the present findings are in line with previous adult data suggesting that the SEE is not a phenomenon specifically based on moral judgment. Like adults, children showed the same answer pattern when the norm that was violated by the side effect was (a paradigmatic case of what is usually taken as) conventional as when it was (a paradigmatic case of what is usually taken as) moral [17]. Although the adult SEE pattern might still be compatible with the claim that the side-effect effect is primarily an effect of the moral status of side effects, and holds for other types of norms only as a derived phenomenon, the analogous pattern found in young children in our study renders such a claim implausible. What these findings thus show is that the SEE in children seems not confined to a narrow class of paradigmatic cases of moral norms, but to norms more generally.

It is an interesting question for future research to which kinds of norms (moral, conventional etc.) the SEE extends in which qualitative and quantitative ways. The present findings tested the theoretically derived claim [13,17] that the SEE exists in qualitatively analogous s across moral and conventional kinds of norms. The results confirm this claim. Yet it is conceivable that on a more fine-grained quantitative level the SEE is not equally sensitive to any kind of norm and that different kinds of norms produce SEEs on different orders of magnitude. While this possibility is not something implied by the theories tested here and thus was not the focus of the present study, it might well be an interesting question for future research in this area. Such more quantified analyses might be an important empirical basis for the development of more fine-grained, information-processing models of the cognitive deep structure of the SEE.

All in all, the present findings show that the SEE is a robust phenomenon already early in development, and is not specific to morality, but extends to both conventional and moral norms. As such, these findings speak in favor of a form of rationality account of the SEE: rather than reflecting a distortion in intentionality judgment brought about by blame or other moral emotions, the SEE is actually based on rational processes of action interpretation [13,17,35]. What future research will need to investigate is which type of rationality account can best explain these and related findings: Is the SEE simply a manifestation of very general principles of diagnostic reasoning and thus extends to all kinds of descriptive and prescriptive norms [17,35] or is it tightly coupled with normative considerations and thus exclusively applies to prescriptive norms[13]?

## Supporting Information

S1 Table Structure of the 4 scenarios. (PDF)
