# Supplementary material for: The Side-Effect Effect in Children Is Robust and Not Specific to the Moral Status of Action Effects
Source: PLoS One. 2015 Jul 28;10(7):e0132933. doi: 10.1371/journal.pone.0132933 (PMC4517779; doi:10.1371/journal.pone.0132933)
Supplement: S1 Table — (PDF) [file pone.0132933.s001.pdf]

S1 File. Structure of the 4 scenarios

Scenario Mouse & Bunny

	Moral norm	Conventional norm
Introduction	--	"Listen, have you ever heard of Filla Land? They have many funny rules there. One of them is that bunnies are **supposed to/ not supposed to** run around the room."
	"Look! Here you see mouse and bunny in a cage. Do you know what happens when you let the mouse out? Bunny automatically comes out too. Shall we take a look?" [*Pressing the space bar animates the slide: the cage door opens and both animals leave the cage*]. "Did you see? Both animals left the cage."	
Scenario	"Look! Here you see mouse and bunny in a cage. And this is Anna. Anna says: "I want to play with the mouse. I will let her out."	
	--	"Anna's sister says: "Here in Filla Land bunnies are **supposed to/ not supposed to** run around the room.
**Side-Effect**	Anna's sister says: "Anna, if you let the mouse out, the bunny automatically comes out too…	
	…. and baby Timmy is **happy/frightened."**	… and will run all around the room."
Control question 1	"What happens when Anna releases the mouse?"	
Control question 2	"And what happens with baby Timmy, when the bunny comes out?"	"And are bunnies supposed to run around the room?"
Indifference statement	Anna says "I don't care what happens with baby Timmy. I just want to play with the mouse."	Anna says "I don't care what the bunny does. I just want to play with the mouse."
Control question 3	"Does Anna care what happens with baby Timmy?"	"Does Anna care what the bunny does?"
**Result**	"Exactly, that's why Anna goes ahead and releases the mouse. And then look, …	
	….baby Timmy is **happy/frightened ."**	…the bunny comes out too and runs around the room."
**Test question**	"Baby Timmy is **happy/frightened**, isn't he?...	"The bunny came out of the cage and is running around the room….
	… Did Anna do this intentionally?	

Horse & Cow

	Moral norm	Conventional norm
Introduction	--	"Listen, have you ever heard of Filla Land? They have many funny rules there. One of them is that cows are **supposed to/ not supposed to** run around the forest."
	"Look! Here you see cow and horse in a stable. Do you know what happens when you let the horse out? Cow automatically comes out too. Shall we take a look?" [*Pressing the space bar animates the slide: the stable door opens and both animals leave the stable*]."Did you see? Both animals left the stable."	
Scenario	"Look! Here you see cow and horse in a stable. And this is Peter. Peter says: "I want to play with the horse. I will let it out."	
	--	Peter's brother says: "Here in Filla Land cows are **supposed to/ not supposed to** run around the forest."
**Side-Effect**	Peter's brother says: "Peter, if you let the horse out, the cow automatically comes out too…	
	…. and Bello the dog is **happy/frightened."**	… and will run all around the forest."
Control question 1	"What happens when Peter releases the horse?"	
Control question 2	"And what happens with Bello the dog when the cow comes out?"	"And are cows supposed to run around the forest?"
Indifference statement	Peter says "I don't care what happens with Bello the dog.<br>I just want to play with the horse."	Peter says "I don't care what the cow does. I just want to play with the horse."
Control question 3	"Does Peter care what happens with Bello the dog?"	"Does Peter care what the cow does?"
**Result**	"Exactly, that's why Peter goes ahead and releases the horse. And then look, the cow comes out too …	
	…and Bello the dog is **happy/frightened ."**	… and runs around the forest."
**Test question**	"Bello the dog is, **happy/frightened**, isn't he?...	"The cow came out of the stable and is running around the forest….
	… Did Peter do this intentionally?"	

Train

	Moral norm	Conventional norm
Introduction	--	"Listen, have you ever heard of Filla Land? They have many funny rules there. One of them is that big trains are **supposed to/ not supposed to** drive into the city."
	"Look! Here you see a big train and a small train. Do you know what happens when you push the start-button? Both trains start driving. Shall we take a look?" [*Pressing the space bar animates the slide: Both trains start driving*]."Did you see? Both trains started driving."	
Scenario	"Look! Here you see a big train and a small train in the train-station. And this is Emil. Emil says: "I want the small train to drive into the city. I will press the start-button."	
	--	Emil's friend says: "Here in Filla Land big trains are **supposed to/ not supposed to** drive into the city.
**Side-Effect**	Emil's friend says: "Emil, if you press the start-button, the big train also starts driving…	
	…. It **drives well/is broken** and then the people on the train are **happy/upset."**	… and drives into the city."
Control question 1	"What happens when Emil pushes the start-button?"	
Control question 2	"And what happens with the people on the big train when it starts driving?"	"And are big trains supposed to drive into the city?"
Indifference statement	Emil says: "I don't care what happens with the people on the big train. I just want the small train to start driving."	Emil says: "I don't care what the big train does. I just want the small train to start driving."
Control question 3	"Does Emil care what happens with the people on the big train?"	"Does Emil care what the big train does?"
**Result**	"Exactly, that's why Emil goes ahead and pushes the start-button. And then look, the big train also starts driving…	
	… and the people on the big train are **happy/upset."**	… and drives into the city."
**Test question**	"The people on the big train are **happy/upset**, aren't they?...	"The big train drives into the city…
	… Did Emil do this intentionally?"	

Streetcar

	Moral norm	Conventional norm
Introduction	--	"Listen, have you ever heard of Filla Land? They have many funny rules there. One of them is that short streetcars are **supposed to/ not supposed to** drive to the lake."
	"Look! Here you see a short and a long streetcar. Do you know what happens when you push the start-button? Both streetcars start driving. Shall we take a look?" [*Pressing the space bar animates the slide: Both streetcars start driving*]. "Did you see? Both streetcars started driving."	
Scenario	"Look! Here you see a short and a long streetcar at the stop. And this is Helga. Helga says: "I want the long streetcar to start driving. I will press the start-button."	
	--	Helga's friend says: "Here in Filla Land short streetcars are **supposed to/ not supposed to** drive to the lake."
**Side-Effect**	Helga's friend says: "Helga, if you press the start-button, the short streetcar also starts driving…	
	…It is **especially comfortable/ missing comfortable seats** and then the people on the streetcar are **happy/upset."**	… and drives to the lake."
Control question 1	"What happens when Helga pushes the start-button?"	
Control question 2	"And what happens with the people on the short streetcar when it starts driving?"	"And are short streetcars supposed to drive to the lake?"
Indifference statement	Helga says: "I don't care what happens with the people on the short streetcar. I just want the long streetcar to start driving."	Helga says: "I don't care what the short streetcar does. I just want the long streetcar to start driving."
Control question 3	"Does Helga care what happens with the people on the short streetcar?"	"Does Helga care what the short streetcar does?"
**Result**	"Exactly, that's why Helga goes ahead and pushes the start-button. And then look, the short streetcar also starts driving…	
	…and the people on the short streetcar are **happy/upset."**	…and drives to the lake."
**Test question**	"The people on the short streetcar are **happy/upset**, aren't they?...	"The short streetcar drives to the lake…
	… Did Helga do this intentionally?"	
